# Supplementary material for: Lift-out cryo-FIBSEM and cryo-ET reveal the ultrastructural landscape of extracellular matrix
Source: J Cell Biol. 2024 Mar 20;223(6):e202309125. doi: 10.1083/jcb.202309125 (PMC10955043; doi:10.1083/jcb.202309125)
Supplement: Table S1 — shows examples of reported dimensions for different ECM fibers. [file JCB_202309125_TableS1.docx]

| Protein | Periodicity/Spacing | Diameter | Reference |
| --- | --- | --- | --- |
| Collagen I fibers | 64-67 nm | 15 to >500 nm | (Ricard-Blum, 2011) |
| Collagen VI fibers | 105 nm  112 nm  85 nm | Not reported  15-19 nm^*^  Not reported | (Furthmayr et al., 1983)  (Baldock et al., 2003)  (Lansky et al., 2019) |
| Fibronectin fibers | 42-280 nm/84 nm mean^**^  ~95 nm^#^ | 6-12 nm  Not reported | (Dzamba and Peters, 1991)  (Früh et al., 2015) |
| FIBRILLIN MICROFIBRILS | 56 nm (resting state)  56.2 nm^***^  65.5 nm^***^  100.3 nm^***^  161.5 nm^***^ | 8-12 nm  20.1 nm^***^  18.3 nm^***^  16.9 nm^***^  12.7 nm^***^ | (Sherratt et al., 2001)  (Glab and Wess, 2008) ^***^ |

**Table S1: Examples of reported dimensions for different ECM fibers.**

Repeat patterns/periodicities/spacings and diameter measurements are listed for papers when measured.

We aimed to provide an overview of several studies reporting the varying measurements for the extracellular matrix filaments. We acknowledge that this table might not be complete, but still underlines the variability of filament assemblies.

* double-bead region

** periodicity was reported to be diameter dependent

# average epitope periodicity

^***^ Glab and Wess (Glab and Wess, 2008) report an increased periodicity and reduced diameter following tissue extension.

**References**

Baldock, C., M.J. Sherratt, C.A. Shuttleworth, and C.M. Kielty. 2003. The supramolecular organization of collagen VI microfibrils. J. Mol. Biol. 330:297–307. 10.1016/S0022-2836(03)00585-0

Dzamba, B.J., and D.M.P. Peters. 1991. Arrangement of cellular fibronectin in noncollagenous fibrils in human fibroblast cultures. J. Cell Sci. 100:605–612. 10.1242/jcs.100.3.605

Glab, J., and T. Wess. 2008. Changes in the molecular packing of fibrillin microfibrils during extension indicate intrafibrillar and interfibrillar reorganization in elastic response. J. Mol. Biol. 383:1171–1180. 10.1016/j.jmb.2008.09.004

Früh, S.M., I. Schoen, J. Ries, and V. Vogel. 2015. Molecular architecture of native fibronectin fibrils. Nat. Commun. 6:7275. 10.1038/ncomms8275

Furthmayr, H., H. Wiedemann, R. Timpl, E. Odermatt, and J. Engel. 1983. Electron-microscopical approach to a structural model of intima collagen. Biochem. J. 211:303–311. 10.1042/bj2110303

Lansky, Z., Y. Mutsafi, L. Houben, T. Ilani, G. Armony, S.G. Wolf, and D. Fass. 2019. 3D mapping of native extracellular matrix reveals cellular responses to the microenvironment. J. Struct. Biol. X. 1:100002. 10.1016/j.yjsbx.2018.100002

Ricard-Blum, S. 2011. The collagen family. Cold Spring Harb. Perspect. Biol. 3:a004978. 10.1101/cshperspect.a004978

Sherratt, M.J., T.J. Wess, C. Baldock, J. Ashworth, P.P. Purslow, C.A. Shuttleworth, and C.M. Kielty. 2001. Fibrillin-rich microfibrils of the extracellular matrix: Ultrastructure and assembly. Micron. 32:185–200. 10.1016/S0968-4328(99)00082-7
