# Supplementary material for: Lift-out cryo-FIBSEM and cryo-ET reveal the ultrastructural landscape of extracellular matrix
Source: J Cell Biol. 2024 Mar 20;223(6):e202309125. doi: 10.1083/jcb.202309125 (PMC10955043; doi:10.1083/jcb.202309125)
Supplement: Table S2 — shows predominant ECM proteins in TIFF CDMs identified by M. Sixt. [file JCB_202309125_TableS2.docx]

|  | Protein | UniProt ID | Sequence Coverage | Peptides  Count | Spectral Count |
| --- | --- | --- | --- | --- | --- |
| 1 | Fibronectin, Isoform 1 | P02751-1 | 76.8 | 346 | 1.155 |
| 2 | Collagen alpha-1(I) chain | P02452 | 79.8 | 268 | 408 |
| 3 | Collagen alpha-2(I) chain | P08123 | 76.4 | 179 | 277 |
| 4 | Collagen alpha-3(VI) chain | P12111 | 77.9 | 347 | 926 |
| 5 | Tenascin | P24821 | 80.9 | 190 | 520 |
| 6 | Collagen alpha-1(VI) chain | P12109 | 68.9 | 92 | 220 |
| 7 | Collagen alpha-2(VI) chain | P12110 | 53.4 | 73 | 195 |
| 8 | TGF-beta-induced protein ig-h3 | Q15582 | 73.8 | 74 | 218 |
| 9 | Galectin-1 | P09382 | 86.7 | 27 | 83 |
| 10 | Galectin-3 | P17931 | 39.2 | 15 | 38 |
| 11 | EMILIN-1 | Q9Y6C2 | 72.8 | 82 | 178 |
| 12 | Fibrillin-1 | P35555 | 62.0 | 235 | 396 |
| 13 | Serpin H1 | P50454 | 63.4 | 31 | 84 |
| 14 | Collagen alpha-1(III) chain | P02461 | 54.7 | 101 | 109 |
| 15 | Fibronectin, Isoform 11 | P02751-11 | 76 | 331 | 1.091 |
| 16 | Pyruvate kinase PKM | P14618 | 73.3 | 40 | 67 |
| 17 | Fibulin-2, Isoform 2 | P98095-2 | 49.5 | 53 | 88 |
| 18 | Collagen alpha-2(V) chain | P05997 | 39.2 | 44 | 48 |
| 19 | Collagen alpha-1(V) chain | P20908 | 29.4 | 47 | 62 |
| 20 | Collagen alpha-1(XII) chain | D6RGG3 | 65.2 | 190 | 277 |
| 21 | Tenascin, Isoform 4 | P24821-4 | 82.7 | 186 | 516 |
| 22 | Decorin | P07585 | 74.1 | 29 | 48 |
| 23 | BM-specific heparan sulfate proteoglycan core protein | P98160 | 66.0 | 214 | 357 |
| 24 | Fibulin-1 | P23142 | 68.4 | 39 | 72 |
| 25 | Fibrillin-2 | P35556 | 48.5 | 136 | 201 |

**Table S2: Predominant ECM proteins in TIFF CDMs identified by MS.**

TIFF CDMs were grown for 14 days and then decellularized and analyzed by LC-MS/MS. Raw data was searched against a *Homo sapiens* reference proteome and filtered for ECM proteins. ECM proteins were then sorted by the normalized log10 of their estimated expression value from highest to lowest and the top 25 hits are shown in this table. For each protein, the sequence coverage, peptide count, and spectral count are listed.
