# Supplementary material for: Lift-out cryo-FIBSEM and cryo-ET reveal the ultrastructural landscape of extracellular matrix
Source: J Cell Biol. 2024 Mar 20;223(6):e202309125. doi: 10.1083/jcb.202309125 (PMC10955043; doi:10.1083/jcb.202309125)
Supplement: Table S3 — shows scouting of different cryoprotectants and buffers for their vitrification potential. [file JCB_202309125_TableS3.docx]

| **Cryoprotectant** | **Degassed** | **Vitrification** | **Background** |
| --- | --- | --- | --- |
| Native | - | Incomplete | None |
| 15% PVP in PBS | - | Incomplete | Acceptable |
| 15% PVP in 0.1 M PB | - | Incomplete | Acceptable |
| 15% BSA in 0.1M PB | - | Incomplete | High |
| 20% Dextran/5% Sucrose in PBS | - | Complete | High |
| 20% Dextran/5% Sucrose in PBS | + | Complete | High |
| 20% Dextran in PBS | + | Incomplete | Acceptable |
| 10% Dextran in PBS | + | Incomplete | Acceptable |
| 10% Dextran in PB | + | Complete | Acceptable |
| 5% Dextran in PB | + | Incomplete | Acceptable |
| Native | + | Incomplete | None |
| 10% BSA in medium | + | Incomplete | Acceptable |
| 20% BSA in medium | + | Complete | High |

**Table S3: Scouting of different cryoprotectants and buffers for their vitrification potential.**

An overview table showing the tested cryoprotectant/buffer combinations, detailing the achieved vitrification status and the introduced background for each combination. The buffers were chosen based on their physiological properties and their common use for biological specimens. Vitrification was judged according to the occurrence of reflections caused by hexagonal ice crystals in the cryo-lift out lamellae. Vitrification was judged as *complete* if no reflections could be detected and as *incomplete* if reflections of any severity were detected. Background was judged as *none* if no cryoprotectants were added, as *acceptable* if structures within the cryo-lift out lamellae were still distinctively visible, and as *high* if the introduced background obscured structures within the cryo-lift out lamellae.
